# Genome Sequences of *Apibacter* spp., Gut Symbionts of Asian Honey Bees

**DOI:** 10.1093/gbe/evy076

**Published:** 2018-04-04

**Authors:** Waldan K Kwong, Margaret I Steele, Nancy A Moran

**Affiliations:** Department of Integrative Biology, University of Texas at Austin

**Keywords:** gut microbiome, honey bee, bumble bee, strain variation, host specificity

## Abstract

Honey bees have distinct gut microbiomes consisting almost entirely of several host-specific bacterial species. We present the genomes of three strains of *Apibacter* spp., bacteria of the Bacteroidetes phylum that are endemic to Asian honey bee species (*Apis dorsata* and *Apis cerana*). The *Apibacter* strains have similar metabolic abilities to each other and to *Apibacter mensalis*, a species isolated from a bumble bee. They use microaerobic respiration and fermentation to catabolize a limited set of monosaccharides and dicarboxylic acids. All strains are capable of gliding motility and encode a type IX secretion system. Two strains and *A. mensalis* have type VI secretion systems, and all strains encode Rhs or VgrG proteins used in intercellular interactions. The characteristics of *Apibacter* spp. are consistent with adaptions to life in a gut environment; however, the factors responsible for host-specificity and mutualistic interactions remain to be uncovered.

## Introduction

Honey bees are critical agricultural pollinators worldwide and have suffered from high rates of colony failure in recent years (Gallai et al. 2009). Worker honey bees harbor distinctive gut bacterial communities that typically consist of fewer than 10 member genera (Kwong and Moran 2016a). These bacteria are important for maintaining proper immune function (Emery et al. 2017; Kwong et al. 2017), gut physiology (Zheng et al. 2017), and nutrient processing (Lee et al. 2015; Kešnerová et al. 2017; Zheng et al. 2017) within bee hosts. The microbiome of the Western honey bee (*Apis mellifera*) has been the attention of many recent studies; however, little is known about the microbiota of its Asian relatives. Two species, the Eastern honey bee (*Apis cerana*) and the giant honey bee (*Apis dorsata*), are widespread throughout South and East Asia. These species have high local economic value as pollinators and as producers of honey and other hive products (Oldroyd and Wongsiri 2006). In particular, *Apis cerana* is kept domestically and accounts for a substantial proportion of the apiculture industry in some nations, including India and China.

Surveys of the gut microbiota of these bees show the presence of bacteria from the phylum Bacteroidetes as resident members (Ahn et al. 2012; Kwong et al. 2017); in contrast, bacteria of this lineage are rarely found in *Apis mellifera*. Strains have been isolated and classified as a novel genus, *Apibacter* (Kwong and Moran 2016b; Praet et al. 2016). In *Apis cerana* and *Apis dorsata*, >80% of adult worker bees are colonized with *Apibacter* (Kwong et al. 2017). *Apibacter* is also found in bumble bees, although with more sporadic occurrence than in the Asian honey bees (Koch and Schmid-Hempel 2011, 2012; Lim et al. 2015; Praet et al. 2016; Kwong et al. 2017). Different bee species appear to harbor different strains of *Apibacter*, suggesting that these bacteria are adapted to specific hosts (Kwong and Moran 2016b; Kwong et al. 2017). In bumble bees, *Apibacter* may be a beneficial symbiont, as it is associated with decreased infection by *Crithidia bombi*, a trypanosomatid gut parasite (Mockler et al. 2017).

Here, we present the genomes of three *Apibacter* strains from the honey bees *Apis cerana* and *Apis dorsata*. Together with a publically available *Apibacter mensalis* strain from a bumble bee, we conduct preliminary genomic analyses to reveal their encoded functions and potential role in the bee gut community.

## Materials and Methods

Cultivation and genomic sequencing of *Apibacter* was conducted as previously described (Kwong and Moran 2016b). Briefly, strains were grown on heart infusion agar (Hardy Diagnostics) supplemented with 5% defibrinated sheep blood, with incubation at 35°C in 5% CO_2_. DNA was extracted for paired-end sequencing on the Illumina MiSeq platform at the Genome Sequencing and Analysis Facility at the University of Texas at Austin (Kwong and Moran 2016b). In total, 2.6 million 300-bp Illumina MiSeq reads were acquired for strains wkB180, wkB301, and wkB309. Reads for strains wkB180 and wkB301 were assembled using MaSuRCA 3.2.2 (Zimin et al. 2013). Assembly of strain wkB309 was performed with Velvet 1.2.10 (Zerbino and Birney 2008) and CLC Genomics Workbench 5.5 (QIAGEN), and improved by mapping reads back onto assembled contigs using Bowtie 2 (Langmead and Salzberg 2012) and manual inspection. This multistep assembly for wkB309 was done to achieve the best possible genome assembly with the available short-read (Illumina) data. The wkB309 genome was selected as it had the highest quality assembly following initial assembly with MaSuRCA 3.2.2 and Velvet 1.2.10 (N50 of 260 kb); the laborious nature of this method precluded its use for all the *Apibacter* genomes. The genome of *Apibacter mensalis* R-53146 was retrieved from GenBank (accession no. LIVM00000000.1).

Genome completeness was estimated with CheckM v1.0.11 (Parks et al. 2015), using the taxonomic-specific workflow with the Bacteroidetes phylum taxon set. It should be noted that a lack of completeness by this metric may also be due to lineage-specific losses of marker genes within *Apibacter*, and not necessarily poor assembly. Gene content prediction and annotation for all four *Apibacter* genomes was done on the Rapid Annotation using Subsystem Technology (RAST) 2.0 platform (Overbeek et al. 2014). Orthologous gene clusters were identified and compared using OrthoVenn (Wang et al. 2015). Single-copy orthologs (1,132 proteins; 401,782 positions) were retrieved using OrthoFinder (Emms and Kelly 2015), aligned with MUSCLE (Edgar 2004), and concatenated to build a phylogenetic tree using the LG amino acid substitution model in RAxML v8 (Stamatakis 2014). Metabolic pathways were predicted with Pathway Tools 21.0 (Karp et al. 2002) and the Kyoto Encyclopedia of Genes and Genomes (KEGG) mapper function on RAST 2.0. Pathway holes were manually inspected to verify completeness. CRISPR arrays were predicted with CRISPRFinder (Grissa et al. 2007). The NCBI Batch CD-Search tool (Marchler-Bauer and Bryant 2004) was used to identify conserved domains in putative secreted proteins, and RAxML v8 was used to build the TssB phylogeny.

Genome sequences have been deposited in GenBank under accession nos. PSZL00000000 (strain wkB309), PSZN00000000 (strain wkB180), and PSZM00000000 (strain wkB301).

## Results and Discussion

### Genome Content

**Fig. 1.**
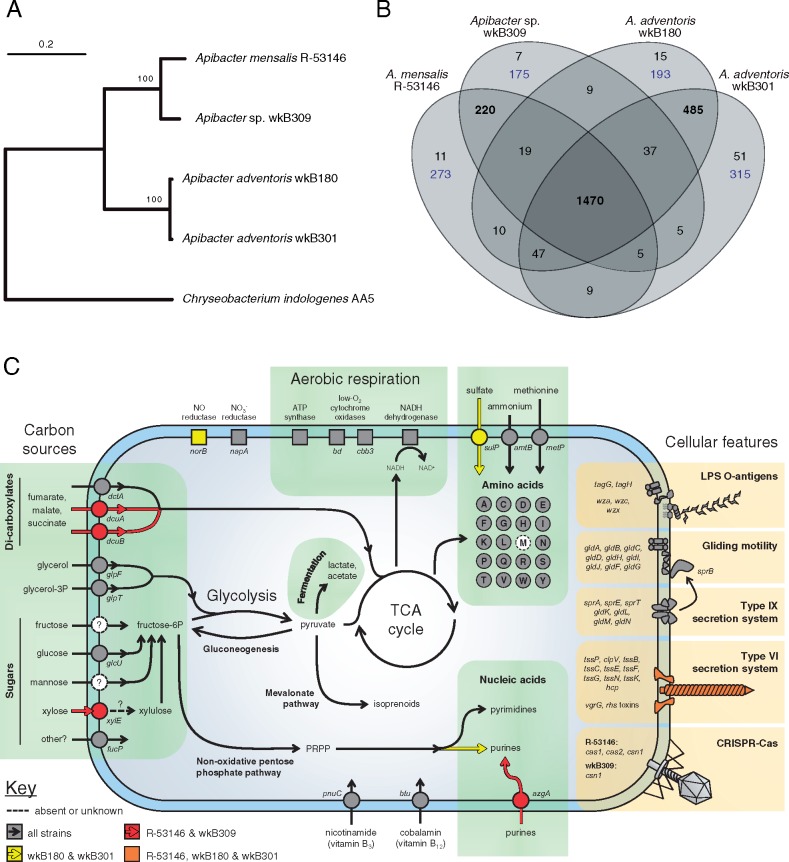
—(*A*) Phylogenetic relationships of sequenced *Apibacter* strains, based on 1,132 single-copy orthologous proteins. Tree was built using the maximum-likelihood algorithm. Bootstrap support (1,000 replicates) indicated at nodes; bar, substitutions per site. *Chryseobacterium indologenes* was used as the outgroup. (*B*) Number of gene clusters (orthologs or paralogs) shared between *Apibacter* strains. Each cluster contains at least 2 genes. Blue text indicates singleton genes unique to a single genome and thus not belonging to orthologous or paralogous clusters. (*C*) Predicted metabolism and cellular features of *Apibacter* spp. Pathways and components colored according to presence across the sequenced strains. Abbreviations: PRPP, 5-phospho-α-d-ribosyl 1-pyrophosphate; LPS, lipopolysaccharide; CRISPR, clustered regularly interspaced short palindromic repeats.

*Apibacter* spp. genomes average 2.5 Mb in size, and have ∼30% G + C content (table 1). The two strains from *Apis dorsata* (wkB180 and wkB301) have larger genomes and lower G + C content than the strains from *Apis cerana* (wkB309) or the bumble bee (R-53146). These differences in genome structure are reflected in their evolutionary relationships, as shown by a whole genome phylogeny (fig. 1*A*). Strains wkB180 and wkB301 are closely related (98.4% ortholog amino acid identity). Strains R-53146 and wkB309 group together, but are more dissimilar (88.3% identity). A large majority of genes are shared by all strains (fig. 1*B*). Three rRNA gene operons are predicted in strain wkB309, the best assembled genome, based on read coverage of contigs. Strain wkB309 carries a single 25.4-kb plasmid, as inferred from the presence of plasmid partitioning and mobilization genes, matching contig end sequences, and higher read coverage for that contig.

**Table 1:** Genome Statistics of *Apibacter* spp.

	*Apibacter mensalis* R-53146	*Apibacter* sp. wkB309	*Apibacter adventoris* wkB180	*Apibacter adventoris* wkB301
**Host**	Red-tailed bumble bee (*Bombus lapidarius*)	Eastern honey bee (*Apis cerana*)	Giant honey bee (*Apis dorsata*)	Giant honey bee (*Apis dorsata*)
**Collection site**	Ghent, Belgium	Genting, Malaysia	Singapore	Kuala Lumpur, Malaysia
**Genome size (bp)**	2,331,098	2,289,083	2,625,614	2,756,245
**G+C mol.**	30.5%	30.6%	28.9%	29.1%
**Plasmids (size)**	–	1 (25.4 kb)	–	–
**Proteins coding genes**	2,097	1,975	2,323	2,550
**tRNAs**	38	36	36	41
**Assembled contigs**	48	22	25	47
**Contig N50 (bp)**	158,456	340,669	165,551	244,991
**Coverage**	302×	130×	75×	100×
**Completeness**^a^	97.95%	97.95%	99.49%	99.49%
**GenBank accession no.**	LIVM00000000	PSZL00000000	PSZN00000000	PSZM00000000

a Completeness estimates based on presence of 286 marker genes common to 419 strains of the phylum Bacteroidetes.

### Metabolism

Based on their genomic content, all strains possess the same core metabolic functionalities (fig. 1*C*), which include the Embden–Meyerhof–Parnas glycolysis pathway, the nonoxidative branch of the pentose phosphate pathway, the ability for gluconeogenesis, and a complete tricarboxylic acid (TCA) cycle. All strains likely use oxidative phosphorylation for energy production, as they encode NADH dehydrogenase and two cytochrome oxidases (*bd* and *cbb_3_*). Genes are present for the de novo synthesis of all proteinogenic amino acids except methionine, for which there is an encoded transporter. Based on their genomic content, pyrimidines can be synthesized de novo; however, only strains wkB301 and wkB180 can produce purines. The genomes also encode pathways for de novo production of several vitamins (pantothenate, tetrahydrofolate, vitamin B6, and likely riboflavin). However, other vitamins or their precursors, including biotin, cobalamin, nicotinamide, and thiamine, need to be imported, as inferred from the absence of genes encoding the necessary biosynthetic enzymes.

*Apibacter* spp. likely subsist on a mix of simple sugars and dicarboxylic acids, based on their gene repertoires. Pathways for glucose, fructose, and mannose degradation are present, which is consistent with in vitro substrate utilization assays (Kwong and Moran 2016b; Praet et. al. 2016). Phosphotransferase systems for carbohydrate import are lacking, which is also the case in some other Bacteroidetes species (Barabote and Saier 2005). Instead, saccharides are likely imported by alternative, or unidentified transporters (fig. 1*C*). The bee gut is a low oxygen environment (Zheng et al. 2017), and this is reflected in how *Apibacter* is predicted to metabolize carbon sources. While the presence of the TCA cycle and NADH dehydrogenase implies a preference for aerobic respiration (Morris and Schmidt 2013), enzymes with activity in low-O_2_ (cytochrome *bd*, cytochrome *cbb_3_*) or anaerobic (*dcuA*, *dcuB*, *glpT*) conditions are also present. If not oxidized in the TCA cycle, substrates broken down by glycolysis are predicted to be fermented to lactate or acetate. Dicarboxylic acids are likely utilized directly by the TCA cycle, in a manner similar to that in *Snodgrassella alvi*, another bee gut symbiont (Kwong et al. 2014).

### Cellular Features

*Apibacter* spp. lack genes encoding flagellum or pilus apparatuses for motility. However, they have a full complement of genes for gliding motility that is typical of Flavobacteriaceae (McBride and Zhu 2013). Strain wkB309 has been observed to glide, and it is likely that all other strains also have this capability (Kwong and Moran 2016b). The type IX secretion system, which is also characteristic of many Bacteroidetes and is necessary for gliding motility (McBride and Nakane 2015; Lasica et al. 2017), is present in all strains.

Bacteria within the bee gut may engage in competitive interactions with each other, using type VI secretion systems (T6SS) to inject toxins into neighboring cells (Steele et al. 2017). Strains R-53146, wkB180, and wkB301 each encode a single T6SS that is related to other Bacteriodetes T6SSs (supplementary fig. S1, Supplementary Material online). All strains encode potential T6SS-secreted proteins: R-53146, wkB180, wkB301, and wkB309 encode 7, 9, 10, and 0 *vgrG*-like genes, respectively, as well as 7, 9, 41, and 4 Rhs-domain containing genes. *S. alvi* and *Gilliamella apicola*, two other Gram-negative members of the bee gut microbial community, also harbor large numbers of Rhs toxin genes (Steele et al. 2017). Further analysis of the genomic organization and predicted functionalities of *Apibacter* T6SSs and putative effectors is presented in supplementary results, Supplementary Material online.

Only *A. mensalis* R-53146 carries a potentially complete CRISPR-Cas system, a bacterial immune mechanism against viruses and foreign DNA (Barrangou et al. 2007). Seven CRISPR spacers were predicted in R-53146, three spacers were in strain wkB309 (which has only the *csn1* gene), while wkB180 and wkB301 each had only a single predicted spacer. Another mechanism to limit the transfer and integration of foreign DNA is through restriction modification (R–M) systems (Wilson and Murray 1991). Each strain has at least one type I R–M system. R–M systems might be shared by members of a coevolving community (Furuta and Kobayashi 2011; Kwong et al. 2014). One R–M system in strain R-53146 shares 97% protein sequence identity with an R–M system in *G. apicola*, suggesting recent transfer of genes between coresident bee gut bacterial species.

## Conclusions

*Apibacter* spp. are microaerobic members of the bee gut community that subsist on a limited set of monosaccharides, dicarboxylates, and glycerol. They are unlikely to participate in the digestion of complex polysaccharides, which stands in contrast to some other bee gut symbionts (e.g., *Gilliamella* and *Bifidobacterium*) as well as gut Bacteroidetes of other animals (e.g., *Bacteroides* and *Prevotella*) (Flint et al. 2008). Based on its physiology, we predict that *Apibacter* colonizes the gut wall, where O_2_ concentration is the highest (Zheng et al. 2017). The encoded type IX secretion system and gliding motility apparatus may help it form biofilms (Kita et al. 2016), perhaps in a similar fashion to *S. alvi*, which is also aerobic and produces biofilms along the gut wall (Martinson et al. 2012; Powell et al. 2016). *Apibacter* co-occurs with *S. alvi* in the Eastern honey bee *Apis cerana*, while the giant honey bee *Apis dorsata* generally lacks *S. alvi* (Kwong et al. 2017). Although the data are sparse, there is possibly an inverse relationship between *Apibacter* and *S. alvi* abundance in bumble bees, perhaps indicating competitive exclusion within a limited ecological niche (Koch and Schmid-Hempel 2012; Lim et al. 2015; Mockler et al. 2017).

The bee gut microbiome is a dynamic community with multiple interacting members. This is reflected in differences among *Apibacter* strains in their complements of encoded accessory elements, including T6SSs, Rhs toxins, CRISPR-Cas systems, and R–M systems. Unlike the core metabolic pathways, these elements probably evolve quickly in response to changes in microbiome composition (such as the presence of bacterial competitors or viruses). Most other noncore genes (i.e., those not shared by all strains, fig. 1*B*) have no known function; those annotated as “hypothetical” constitute 30% of total predicted genes. Closer scrutiny of these strain-specific genes is needed to help uncover the factors responsible for *Apibacter*’s ability to colonize and interact with specific bee hosts.

## Supplementary Material

Supplementary data are available at *Genome Biology and Evolution* online.

## Authors’ Contributions

W.K.K. and N.A.M. designed the study. W.K.K. and M.I.S. analyzed and interpreted the data. W.K.K. drafted the manuscript. All authors read, edited, and approved the final manuscript.

## Supplementary Material

Supplementary Data
