# Supplementary material for: Genome Sequences of Apibacter spp., Gut Symbionts of Asian Honey Bees
Source: Genome Biol Evol. 2018 Apr 4;10(4):1174–9. doi: 10.1093/gbe/evy076 (PMC5913662; doi:10.1093/gbe/evy076)
Supplement: Supplementary Data [file evy076_supp.docx]

**Supplementary data**

**for**

**Genome sequences of *Apibacter* spp., gut symbionts of Asian honey bees**

Waldan K. Kwong, Margaret I. Steele, Nancy A. Moran

**The T6SS locus in *Apibacter***

*A. adventoris* wkB180, *A. adventoris* wkB301, and *A. mensalis* R-53146 each encode one T6SS, which is absent in *Apibacter* sp. wkB309. A maximum likelihood phylogeny of the TssB protein, a well-conserved component of the T6SS contractile sheath, places the *Apibacter* T6SSs in the same clade as the *Bacteroides* T6SSs, suggesting that this complex is quite different from the T6SS found in bee-associated Proteobacteria (Fig. S1). The T6SS loci from wkB180 and wkB301 has 98.82% nucleotide identity to each other, and are fairly similar to the T6SS locus in *A. mensalis* R-53146 (~74% nucleotide identity). These T6SSs have shared synteny and are comprised of 14 genes, including 10 core T6SS genes (*tssP*, *clpV*, *tssB*, *tssC*, *tssE*, *tssF*, *tssG*, *tssN*, *tssK*, and *hcp*), 3 hypothetical genes, and a putative lipoprotein. The *Apibacter* T6SS loci appear to be missing the core T6SS genes *tssQ*, *tssO*, and *tssR*. Searches using BLAST for *Apibacter* proteins homologous to the *Bacteroides fragilis* 638R T6SS proteins, failed to locate the missing genes. Similarly, manual inspection of the region surrounding the T6SS loci did not identify the missing genes: the T6SS locus in all three genomes terminates with a *hcp* gene located at the 3’ end. However, the putative lipoprotein encoded by the *Apibacter* T6SS loci might be *tssO*, which serves the same function in *Bacteroides* as the lipoprotein *tssJ* in Proteobacteria (Coyne et al. 2016). The putative lipoprotein gene in question co-occurs with *tssG* in *Flavobacterium johnsoniae* UW101 as well as in the *Apibacter* genomes, suggesting that this gene is a conserved component of the T6SS.

**VgrG genes in *Apibacter***

# VgrG is a core component of the T6SS that forms the tip of the needle-like structure used to puncture the membranes of target cells. VgrG proteins play a key role in toxin delivery during T6SS-mediated antagonism: some VgrGs have C-terminal toxin domains, while others deliver toxins by binding to a proline-alanine-alanine-arginine (PAAR) domain within the toxin (Bondage et al. 2016). Strains R-53146, wkB180, and wkB301 encode 7, 9, and 10 *vgrG*-like genes, respectively. These *vgrG* genes are diverse in their size and composition, encoding proteins that range from 266 to 1,037 amino acids in length. The longest of these appear to be “evolved” VgrG proteins (Pukatzki et al. 2007), with the VgrG domain comprising only the first half of the protein (the N-terminus). The C-terminus of these proteins has no conserved domains, but is likely to be a toxin fused to VgrG for delivery via the T6SS.

# Because the delivered effectors are sometimes encoded by genes adjacent to *vgrG* (Bondage et al. 2016; Russell et al. 2013), we also examined the genes downstream of each *Apibacter* *vgrG* gene. A high proportion of genes encoding smaller VgrG proteins occur in close proximity to hypothetical genes with PAAR or PAAR-like DUF4280 domains (Rigard et al. 2016), which associate with VgrG to form the piercing spike at the end of the T6SS needle. Diverse toxin genes are also found downstream of the *Apibacter* T6SS, including DNases, RNases, peptidases, and Rhs-family toxins. Some *vgrG* and *vgrG*-associated downstream genes are present in multiple strains, while others are found in only one strain. A few *vgrG* genes appear to be shared between *Apibacter* strains but have different C-terminal sequences and downstream genes, which is consistent with the observation that the C-terminal domain of VgrG determines which effectors are delivered by that particular protein (Bondage et al. 2016). Furthermore, many *vgrG* genes are located in close proximity to genes encoding the Hcp protein that forms the T6SS needle shaft.

# Rhs toxins in *Apibacter*

Rhs proteins are large, polymorphic toxins that function as T6SS effectors in many Gram-negative bacteria. These proteins typically have a conserved core region, containing the YD-repeats characteristic of this protein family, and variable C-terminal toxin domains (Koskiniemi et al. 2013). Rhs toxins in *Apibacter* were identified using the Batch CD-Search tool (Marchler-Bauer et al. 2017) to identify proteins with Rhs domains. Strains R-53146, wkB180, and wkB301 respectively encode 7, 9, and 41 Rhs toxins. Surprisingly, although *Apibacter* sp. wkB309 does not encode a T6SS, it does encode 4 genes containing Rhs domains. Many of the Rhs genes in wkB301 are clusters of fragments found in close proximity to one or more integrase-like genes. Of the 67 Rhs toxins encoded by these strains, only 9 are over 1,000 amino acids in length and more than half of these are encoded by wkB180. Alignment of the Rhs proteins reveals that the majority have truncated N-termini, while a much smaller percentage are missing the C-terminal toxin domain. Furthermore, Rhs toxins with very similar N-terminal domains may have very different toxin domains. For example, half of the Rhs toxins encoded by wkB301 have the same N-terminal sequence, but very few share the same C-terminus. Several Rhs N-termini are present in multiple strains, while others only occur once. Four of the Rhs toxins encoded by R-53146 share the same N-terminus, a sequence that is not present in any other *Apibacter* strain. Similarly, C-terminal toxin domains are sometimes shared between strains. wkB180 and wkB301 have the largest number of shared toxin domains, but toxin domains are also shared between wkB301 and R-53146 and between wkB309 and R-53146.

# Several of the Rhs toxins identified in *Apibacter* contain an SpvB domain, which is characteristic of toxins secreted through the TcdB/TcaC pathway (Zhang et al. 2012), suggesting that a subset of these toxins may not be T6SS effectors. Strain wkB180 encodes two proteins approximately 3,300 amino acids long that contain SpvB, TcdB, and Rhs domains, and are very similar to a protein encoded by wkB301. The most significant difference between these three proteins is the C-terminal region: one of the toxins in wkB180 shares a C-terminal domain with an N-terminally truncated gene in wkB301, while the toxin encoded by wkB301 has a C-terminus containing a serralysin-like zinc metallopeptidase domain. Strains R-53146, wkB180, and wkB309 each encode one protein approximately 2,200 amino acids in length containing SpvB, VCBS, and Rhs domains, which likewise differ primarily in the C-terminus. The protein encoded by *A*. *mensalis* R-53146 has a C-terminal Ntox33 domain, a predicted RNase toxin domain usually exported by the type II secretion system (Zhang et al. 2012). The SpvB domain in *Apibacter* aligns to the N-terminus of the *Salmonella enterica* SpvB protein, which is similar to the N-terminal domain TcaC (part of the secreted insecticidal toxin from *Photorhabdus* *luminescence*) and is required for the secretion of SpvB in *Salmonella* (Gotoh et al. 2003). However, these strains do not encode homologs to the other components of the *S. enterica* SpvABC or *Photorhabdus* TcdAB and TcaC secretion pathways. Interestingly, each gene encoding a protein with a TcdB domain is preceded by a hypothetical gene encoding a 1,023 to 1,032 amino acid protein with a Por secretion system C-terminal sorting domain (TIGR04183), but no other conserved domains. Por (also known as the type IX secretion system), is a component of Bacteroidetes gliding motility, but may also secrete proteins for other functions (Lasica et al. 2017).

# Rhs toxins in *Apibacter* have diverse C-terminal toxin domains. Some of these toxin domains are dissimilar to previously defined toxins, but others have hits against the NCBI Conserved Domains database. In a few cases, the cognate immunity gene – located immediately downstream of the toxin – contains a conserved domain that makes it possible to infer the function of the toxin. These toxin domains include nuclease toxins, such as HNH, LHH, AHH, EHHH, and URI2 (inferred from the presence of Imm12 downstream); RNase toxins, including Ntox33 and Ntox47; lipases domains (with potential immunity genes encoding ankyrin repeats); and peptidases, including the metallopeptidase toxin 4 domain, serralysin-like ZnMc domain, and M91 family metallopeptidase domain. Rhs toxins secreted through T6SSs sometimes have N-terminal PAAR domains, which interact with VgrG. However, we identified only two proteins encoded by *Apibacter* that have PAAR domains. One is a Rhs-family toxin that is 1,540 amino acids in length with a N-terminal PAAR domain and a predicted C-terminal nuclease (LHH) domain encoded by wkB180. The other is a Rhs fragment encoded by wkB301 comprised of little more than the PAAR domain.

# References

# Bondage DD, Lin JS, Ma LS, Kuo CH, Lai EM. 2016. VgrG C terminus confers the type VI effector transport specificity and is required for binding with PAAR and adaptor-effector complex. *Proc Natl Acad Sci U S A*. 113(27):E3931-40.

# Coyne MJ, Roelofs KG, Comstock LE. 2016. Type VI secretion systems of human gut Bacteroidales segregate into three genetic architectures, two of which are contained on mobile genetic elements. *BMC Genomics*. 17:58.

# Gotoh H, Okada N, Kim YG, Shiraishi K, Hirami N, Haneda T, Kurita A, Kikuchi Y, Danbara H. 2003. Extracellular secretion of the virulence plasmid-encoded ADP-ribosyltransferase SpvB in *Salmonella*. *Microb Pathog*. 34(5):227–238.

# Koskiniemi S, Lamoureux JG, Nikolakakis KC, t'Kint de Roodenbeke C, Kaplan MD, Low DA, Hayes CS. 2013. Rhs proteins from diverse bacteria mediate intercellular competition. *Proc Natl Acad Sci U S A*. 110(17):7032–7037.

# Lasica AM, Ksiazek M, Madej M, Potempa J. 2017. The type IX secretion system (T9SS): Highlights and recent insights into its structure and function. *Front Cell Infect Microbiol*. 7:215.

# Marchler-Bauer A, Bo Y, Han L, He J, Lanczycki CJ, Lu S, Chitsaz F, Derbyshire MK, Geer RC, Gonzales NR, Gwadz M, Hurwitz DI, Lu F, Marchler GH, Song JS, Thanki N, Wang Z, Yamashita RA, Zhang D, Zheng C, Geer LY, Bryant SH. 2017. CDD/SPARCLE: functional classification of proteins via subfamily domain architectures. *Nucleic Acids Res*. 45(D1):D200–D203.

# Pukatzki S, Ma AT, Revel AT, Sturtevant D, Mekalanos JJ. 2007. Type VI secretion system translocates a phage tail spike-like protein into target cells where it cross-links actin. *Proc Natl Acad Sci U S A*. 104(39):15508–15513.

# Rigard M, Bröms JE, Mosnier A, Hologne M, Martin A, Lindgren L, Punginelli C, Lays C, Walker O, Charbit A, Telouk P, Conlan W, Terradot L, Sjöstedt A, Henry T. *Francisella tularensis* IglG Belongs to a Novel Family of PAAR-Like T6SS Proteins and Harbors a Unique N-terminal Extension Required for Virulence. *PLoS Pathog*. 12(9):e1005821.

# Russell AB, LeRoux M, Hathazi K, Agnello DM, Ishikawa T, Wiggins PA, Wai SN, Mougous JD. 2013. Diverse type VI secretion phospholipases are functionally plastic antibacterial effectors. *Nature*. 496(7446):508–512.

# Zhang D, de Souza RF, Anantharaman V, Iyer LM, Aravind L. 2012. Polymorphic toxin systems: Comprehensive characterization of trafficking modes, processing, mechanisms of action, immunity and ecology using comparative genomics. *Biol Direct*. 25;7:18.

#
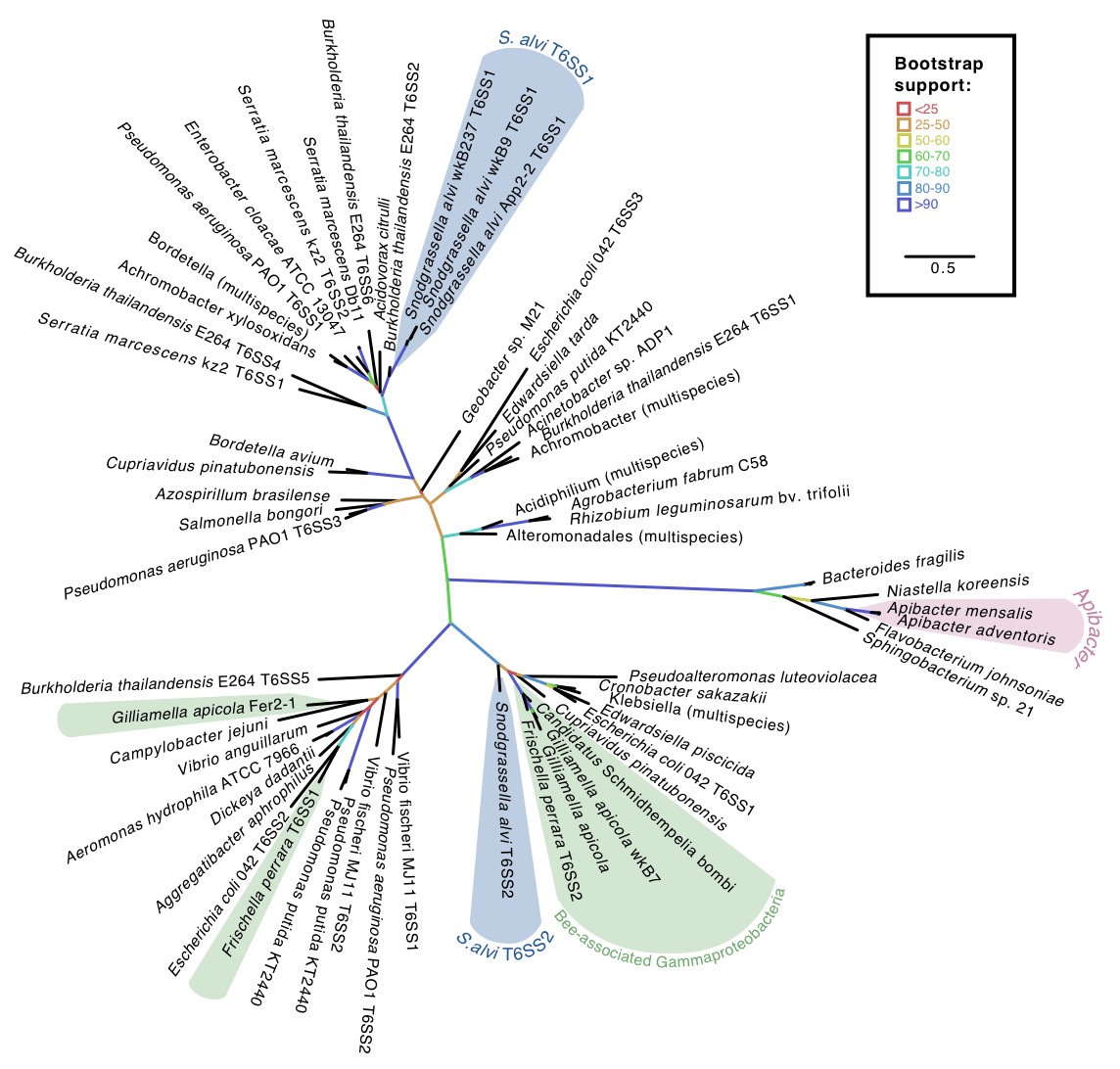


**Figure S1.** Maximum likelihood phylogeny of *Apibacter* T6SSs, inferred from TssB protein.
